# Methylation-Driven Gene PLAU as a Potential Prognostic Marker for Differential Thyroid Carcinoma

**DOI:** 10.3389/fcell.2022.819484

**Published:** 2022-01-24

**Authors:** Min Wu, Bo Wei, Sai-Li Duan, Mian Liu, Deng-Jie Ou-Yang, Peng Huang, Shi Chang

**Affiliations:** ^1^ Department of General Surgery, Xiangya Hospital Central South University, Changsha, China; ^2^ National Clinical Research Center for Geriatric Disorders, Xiangya Hospital, Changsha, China; ^3^ Clinical Research Center for Thyroid Disease in Hunan Province, Changsha, China; ^4^ Hunan Provincial Engineering Research Center for Thyroid and Related Diseases Treatment Technology, Changsha, China

**Keywords:** differential thyroid carcinoma, prognosis, methylation-driven gene, PLAU, immune responce

## Abstract

**Purpose:** Aberrant DNA methylation plays a crucial role in the tumorigenesis of differentiated thyroid cancer (DTC); nevertheless, the factors leading to the local and regional recurrence of DTC are not well understood. This study aimed to establish the connection between DNA methylation-driven genes and the recurrence of DTC.

**Methods:** RNA sequencing profiles and DNA methylation profiles of DTC were downloaded from The Cancer Genome Atlas (TCGA) database. Combined application of the methylmix R package and univariate Cox regression analyses were used to screen and distinguish prognosis-related methylation-driven genes. Multivariate Cox regression analyses were utilized to identify the target genes that were closely associated with the recurrence of DTC. Then, correlations between the expression levels of the target genes and the clinicopathological features were verified, as well as their potential biological functions.

**Results:** A total of 168 Methylation-driven genes were differentially expressed in thyroid cancer, among which 10 genes (GSTO2, GSTM5, GSTM1, GPX7, FGF2, LIF, PLAU, BCL10, SHARPIN and TNFRSF1A) were identified as Hub genes. We selected PLAU for further analysis because PLAU was most strongly correlated with DTC recurrence and the DNA methylation levels of PLAU were closely associated with multiple clinicopathological features of DTC. PLAU was significantly upregulated in DTC, and patients with a high expression level of PLAU had a higher risk of recurrence (*p* < 0.05). Functional predictions suggested that PLAU-related genes were mainly involved in the regulation of immune-related signaling pathways. Moreover, the mRNA level of PLAU was found to be positively correlated with the cell markers of neutrophils and dendritic cells. In addition, we found that two DNA methylation sites (cg06829584, cg19399285) were associated with abnormal expression of PLAU in DTC.

**Conclusion:** The methylation-driven gene PLAU is an independent risk factor for the recurrence of DTC and it functions as an oncogene through the regulation of immune-related signaling pathways, which offers new insight into the molecular mechanisms of thyroid cancer and provides new possibilities for individualized treatment of thyroid cancer patients.

## Introduction

Over the past 4 decades, the incidence of thyroid cancer in the United States has increased by 3% per year, primarily due to an increase in papillary and follicular thyroid cancers, which are also known as differentiated thyroid cancer (DTC) (Kitahara and Sosa, 2016; Lim et al., 2017). Comprehensive treatment based on surgery can result in a 10-years survival rate of >95% for patients with thyroid cancer (Haugen et al., 2016). However, because of the low mortality and longer survival of DTC patients, some patients experience recurrences, which increase morbidity and reduce their quality of life. Persistent structural disease or recurrence was found in approximately 3% of low-risk patients, 21% of intermediate-risk patients, and 68% of high-risk patients (*p* < 0.001) (Mazzaferri and Jhiang, 1994; Tuttle et al., 2010; Force et al., 2017). Optimizing the initial treatment requires consideration of the risk of recurrence after initial conservative surgery versus the potentially impaired quality of life due to the higher rates of complications associated with more aggressive surgery (including hypoparathyroidism and recurrent laryngeal nerve injury) (Wang and Sosa, 2018). Therefore, accurate preoperative assessment and risk stratification for recurrence are the first critical steps in optimizing the management of thyroid nodules and DTC (Wang and Sosa, 2018). Studies have shown that older age, initial central and lateral lymph node metastasis, and multifocal tumors are independent risk factors for thyroid cancer recurrence (Kim et al., 2017). Despite significant advances in survival prediction and recurrence prediction in the AJCC eighth edition, the criteria remain unsatisfactory due to individual heterogeneity (Gan and Randle, 2019). Therefore, identifying more effective biomarkers that can distinguish patients with a poor prognosis is necessary.

DNA methylation, one of the epigenetic mechanisms, can determine the cellular state through its regulation of transcription (Dawson and Kouzarides, 2012; Köhler and Rodríguez-Paredes, 2020). Increasing evidence indicates that aberrant DNA methylation is a significant feature of cancer cells, and can be used as a biomarker for tumor diagnosis, prognosis and prediction (Koch et al., 2018). Overall hypomethylation and hypermethylation of promotor CGIs (CpG islands) represent signature methylation changes in cancer and they are involved in tumorigenesis (Ehrlich, 2002; Koch et al., 2018). DNA methylation is an important epigenetic mechanism in the occurrence and development of thyroid cancer, and it has great potential in diagnosis and treatment. Studies have shown that some genes such as p16INK4A and MLH1, are frequently hypermethylated in many cancers, including thyroid cancer (Schagdarsurengin et al., 2006). In thyroid cancer, most DNA methylation alterations occur outside the promoter regions, most of which are hypomethylations and are specifically associated with tumor histology (Zafon et al., 2019). In addition, some studies have shown that DNA methylation can regulate the function of the immune system at the transcriptional level, and global DNA demethylation could reduce tumor immunity and thus affect the development of tumors, promote immune evasion, and undermine the benefit of immunotherapy (Jung et al., 2019; Morales-Nebreda et al., 2019). Moreover, the methylation of certain genes is related to lymph node metastasis and the high invasiveness of tumors (Nagata et al., 2017; Wu et al., 2017). Chen constructed a nomogram that combined both 6-DNA methylation signatures to predict the recurrence-free survival of PTC(Chen et al., 2020). However, this study focused on the DNA methylation signature, and which did not provide a potential target for therapeutic action.

To identify biomarkers with diagnostic and prognostic value that could be potential therapeutic targets for DTC, we analyzed related methylation and transcription profiles of DTC through The Cancer Genome Atlas (TCGA) program and GEO database. Besides, we explored the methylation-driven genes’ expression level among various clinicopathological subgroups and their possible mechanisms in DTC.

## Materials and Methods

### Patients and Samples

A total of 572 RNA sequencing profiles (513 THCA samples and 59 nontumor samples) and 571 DNA methylation profiles (515 THCA samples and 56 nontumor samples) were obtained from the UCSC Genome Browser (http://xena.ucsc.edu/) (through December 19, 2020). The corresponding clinical information for patients with differentiated thyroid cancer (Supplementary Table S1) was obtained from Cbioportal (https://www.cbioportal.org/). Of the 571 DTC samples for which DNA methylation data were available, 568 DTC samples included both RNA sequencing data and paired DNA methylation data. Of the 512 tumor samples, only 507 included disease-free survival time and recurrence status. The TCGA cohort gene expression data were obtained through the Illumina HiSeq 2000 RNA sequencing platform, while the DNA methylation data were obtained using the Illumina Infinium Human Methylation 450 platform. The DNA methylation values for all CpG sites in the promoter of each gene (transcription start site (TSS) 1500 and TSS 200) were calculated as the DNA methylation values for that gene. The GSE86961 dataset (consisting of 41 normal and 41 tumor samples) and the GSE97466 dataset (consisting of 50 normal and 74 tumor samples) were used to validate the methylation levels of the methylation driver genes. Correspondingly, GSE33630 (including 45 normal and 60 tumor samples), GSE35570 (including 51 normal and 65 tumor samples) and GSE60542 (including 30 normal and 33 tumor samples) were used to validate the transcriptional expression levels of the methylation driver genes. The validation datasets were all obtained from the GEO database (https://www.ncbi.nlm.nih.gov/geo/). The present study obtained ethics approval from the Ethics Committee of Xiangya Hospital Central South University (2019030440). We collected 12 pairs of matched cancerous and para-cancerous tissues from DTC patients at Xiangya Hospital Central South University, validation of the targeting genes was performed via immunohistochemical staining according to the instruction in the kit.

### Methylation-Driven Gene Identification

MethylMix is a popular enrichment algorithm, which was extensively utilized in medical studies (Liu et al., 2021a; Liu et al., 2021b; Liu et al., 2021c; Liu et al., 2021d). The MethylMix package in R was used to identify DNA methylation events that significantly affected the expression of the corresponding gene, indicating that the gene is affected by DNA methylation (Cedoz et al., 2018). The MethylMix analysis consisted of three parts. First, correlation analysis of gene expression data and paired methylation data from DTC samples was used to identify DNA methylation events that lead to changes in gene expression. Methylation-driven genes with R < −0.3 were used for further analysis. The beta mixture model was then used to define the methylation status of the patients, excluding the need for arbitrary thresholds. Finally, the Wilcoxon rank sum test was used to compare the DNA methylation status between 512 DTC samples and 56 corresponding nontumor samples. Multiple iterations of the test were performed based on *p* < 0.05 (Cedoz et al., 2018).

### Hub Gene Screening *via* MCODE and K-Means Clustering

The MCODE algorithm of the Metascape tool (https://metascape.org/) can set parameters based on the data, thus creating an arbitrary number and the size of subnets of the same cluster to identify the critical components of the key nodes (Magraner-Pardo et al., 2021; Tian et al., 2021). Based on the MCODE module, we first obtained several key subnetworks. Based on the STRING functional protein association network and its built-in Kmean clustering function, we further explored the association of the MCODE module genes at the protein expression level and obtained a combined score (Jayaraman et al., 2015; Kumar et al., 2017).

### Survival Analysis

To assess the prognostic value of the subnetwork genes, we analyzed the relationship between the gene expression levels and recurrence-free survival using the custom data module from KMplotter (http://kmplot.com/analysis/). Genes with *p* < 0.05 were included in the multifactorial Cox regression, where genes with the highest HR values were selected for subsequent analysis. We also analyzed the prognostic value of the gene-matched CpG probes.

### Clinical Relevance Analysis

Based on the methylation levels of the target genes, we divided the tumor samples into hypermethylated and hypermethylated groups. The methylation levels of the target genes were then analyzed in relation to the patient age, sex, histological stage, AJCC stage, lymph node metastasis status, distant metastasis status and BRAF mutation status. The AJCC stage of the TCGA samples was converted to the 8th edition stage according to the latest guidelines. Correspondingly, we also analyzed the correlation between the transcript levels of the target genes and the patients’ clinicopathological characteristics.

### Functional and Pathway Enrichment Analysis

We first submitted the list of methylation driver genes to Metascape and analyzed their GO and KEGG enrichment results (Chen et al., 2021). After selecting the target genes, we also analyzed the KEGG enrichment results of the target genes with their associated differentially expressed genes. The corresponding results were also validated using GSEA (Subramanian et al., 2007).

### Immune-Related Analysis

The results of the pathway enrichment suggest that both methylation driver and target genes are associated with immune responses and immune pathway activation. We therefore proceeded to correlate the target genes with the immune components of the thyroid cancer microenvironment. The Tumor Immunity Estimation Resource (TIMER, available from http://cistrome.org/TIMER) was used to examine the extent to which the six immune cells of the target gene, including B cells, CD4^+^ T cells, CD8^+^ T cells, neutrophils, macrophages, and natural killer cells, were correlated in the thyroid cancer samples from TCGA (Li et al., 2017). For the highly correlated immune cells (|R|>0.5), the final results presented were corrected for tumor purity (Rhee et al., 2018).

### Statistical Analysis

*p* values < 0.05 were considered significantly different if not specifically mentioned. Correlation analysis was performed by linear regression analysis. The chi-square test was used for clinical correlation. Statistical analyses were completed using R v.3.6.1 software.

## Results

### Identification of Methylation-Driven Genes in Thyroid Carcinoma

**FIGURE 1:**
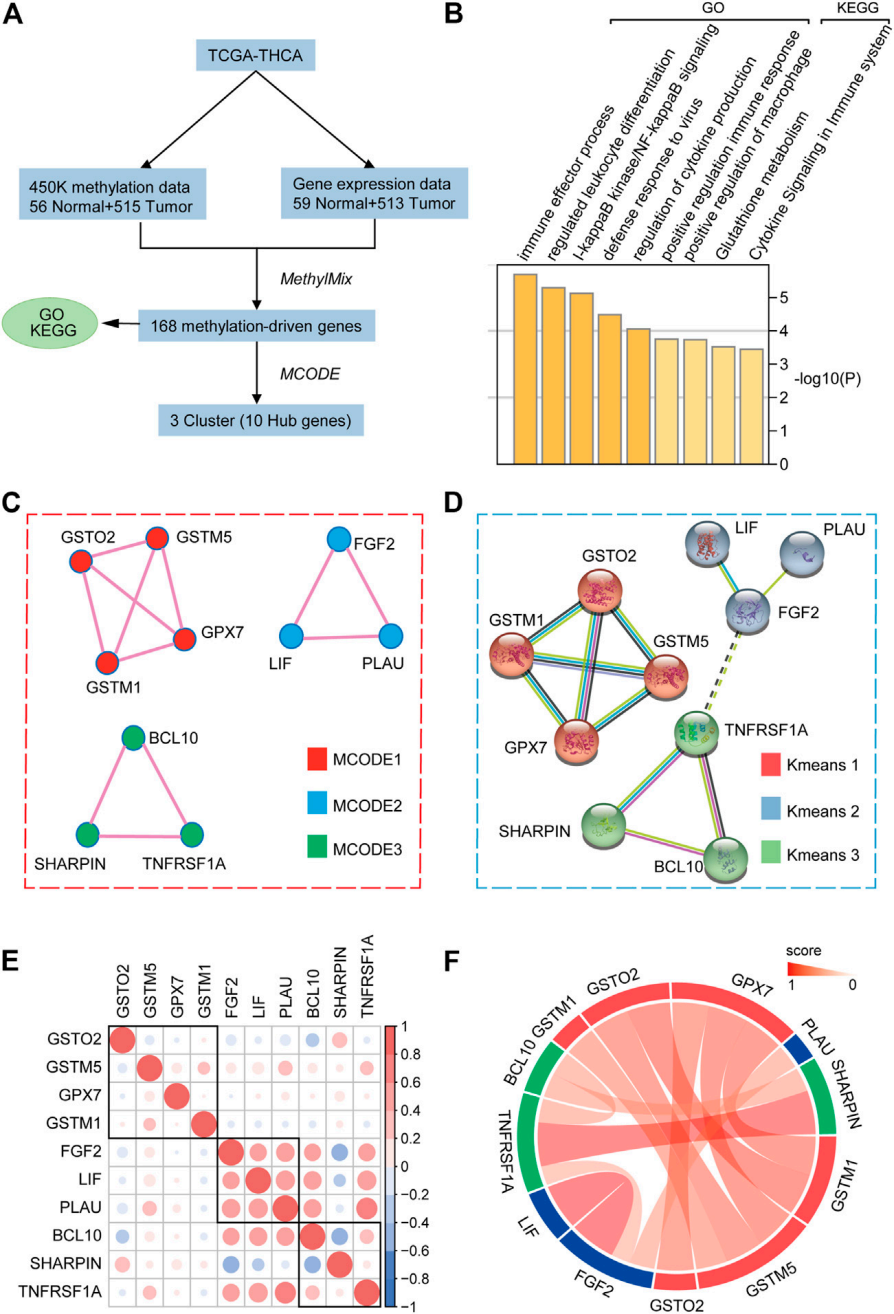
Selection of hub methylation-driven genes. **(A)** Schematic workflow for the screening of methylation-driven genes in THCA. **(B)** GO and KEGG pathway analyses of 168 indicated methylation-driven genes. **(C)** MCODE algorithm identifying the major modules of 168 methylation-driven genes. **(D)** Protein-protein interaction network and K-means clustering model of hub genes. **(E)** Hub gene correlation at the transcriptional level. **(F)** Hub genes’ linkage score at the protein level.

The flow chart of the exploration of methylation-driven genes is shown in Figure 1A. Our study included DNA methylation data from 571 samples from thyroid carcinoma patients, including 56 normal samples and 515 tumor samples. The gene expression data were gathered from 572 thyroid carcinoma specimens, including 59 normal samples and 513 tumor samples. Abnormal methylation expression and gene expression data were extracted and analyzed, and we derived 168 DNA methylation driver genes with differential expression whose biological function is related to immunity. The GO analysis results indicated that 168 methylation-driven genes were significantly enriched in “immune effector process”, “regulated leukocyte differentiation”, and “I-kappaB kinase/NF-kappaB signaling”. The KEGG pathway analysis revealed that they were mainly associated with “positive regulation of macrophages”, “glutathione metabolism”, and “cytokine signaling in immune system” (Figure 1B). Interestingly, MCODE identified three important subnetworks consisting of 10 points (Figure 1C and Supplementary Figure S1). Kmeans clustering from the STRING website suggested that Hub genes showed similar clustering results at the protein level, indicating a stable linkage between Hub genes (Figure 1D). In addition, we verified the correlation at the transcriptional level (Figure 1E) and the linkage score at the protein level (Figure 1F) among the Hub genes.

### Aberrant PLAU Methylation in Thyroid Cancer Is Closely Associated With Clinicopathological Features

**FIGURE 2:**
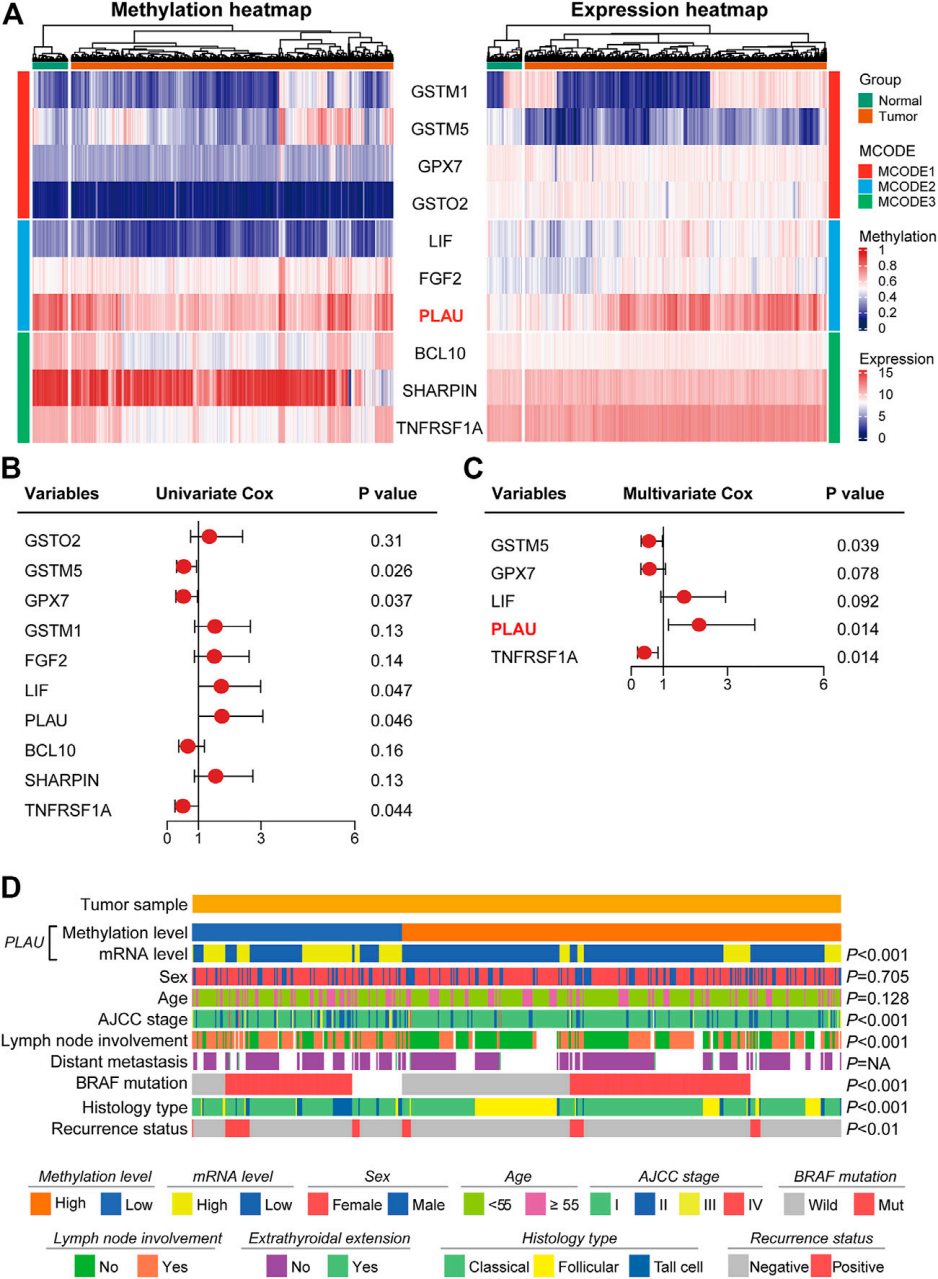
Methylation levels of PLAU correlated with the clinicopathological characteristics in THCA patients. **(A)** Heatmap showing the methylation profile and transcriptome profile of ten Hub genes. **(B–C)** Univariate Cox regression and multivariate Cox regression analysis identified PLAU as a significant risk factor for recurrence. **(D)** Association of the methylation levels of PLAU with the clinicopathological features in THCA patients.

We further screened for hub genes and identified the genes associated with the clinicopathological features of DTC. The heatmap shows the methylation levels and the corresponding transcript levels of the Hub gene in thyroid cancer and the adjacent normal tissues (Figure 2A). Among the 10 hub genes, PLAU showed the most obvious differences in methylation levels (hypermethylation in the tumor tissue) and transcript levels (high expression in tumor tissue). To investigate the correlation between the expression levels and the risk of PTC recurrence, the hub gene with *p* < 0.05 in the log-rank analysis was included in the multivariate Cox regression analysis (Figure 2B). Multivariate Cox regression analysis showed that high PLAU expression was an independent risk factor for thyroid cancer recurrence (HR = 2.113, *p* = 0.014) (Figure 2C). Therefore, PLAU was singled out as a starting point for subsequent analysis.

Then, we explored the association between the methylation level of PLAU and clinical variables in tumor specimens. We found that the methylation level of PLAU was closely related to the AJCC stage (*p* < 0.001), lymph node involvement (*p* < 0.001), BRAF mutation (*p* < 0.001), histology type (*p* < 0.001) and recurrence status (*p* < 0.01). In comparison, other clinical items, such as sex (*p* = 0.705), age (*p* = 0.128) and distant metastasis (*p* = NA) had no association with the methylation level of PLAU (Figure 2D).

### Validation of the PLAU Methylation and Transcript Levels

**FIGURE 3:**
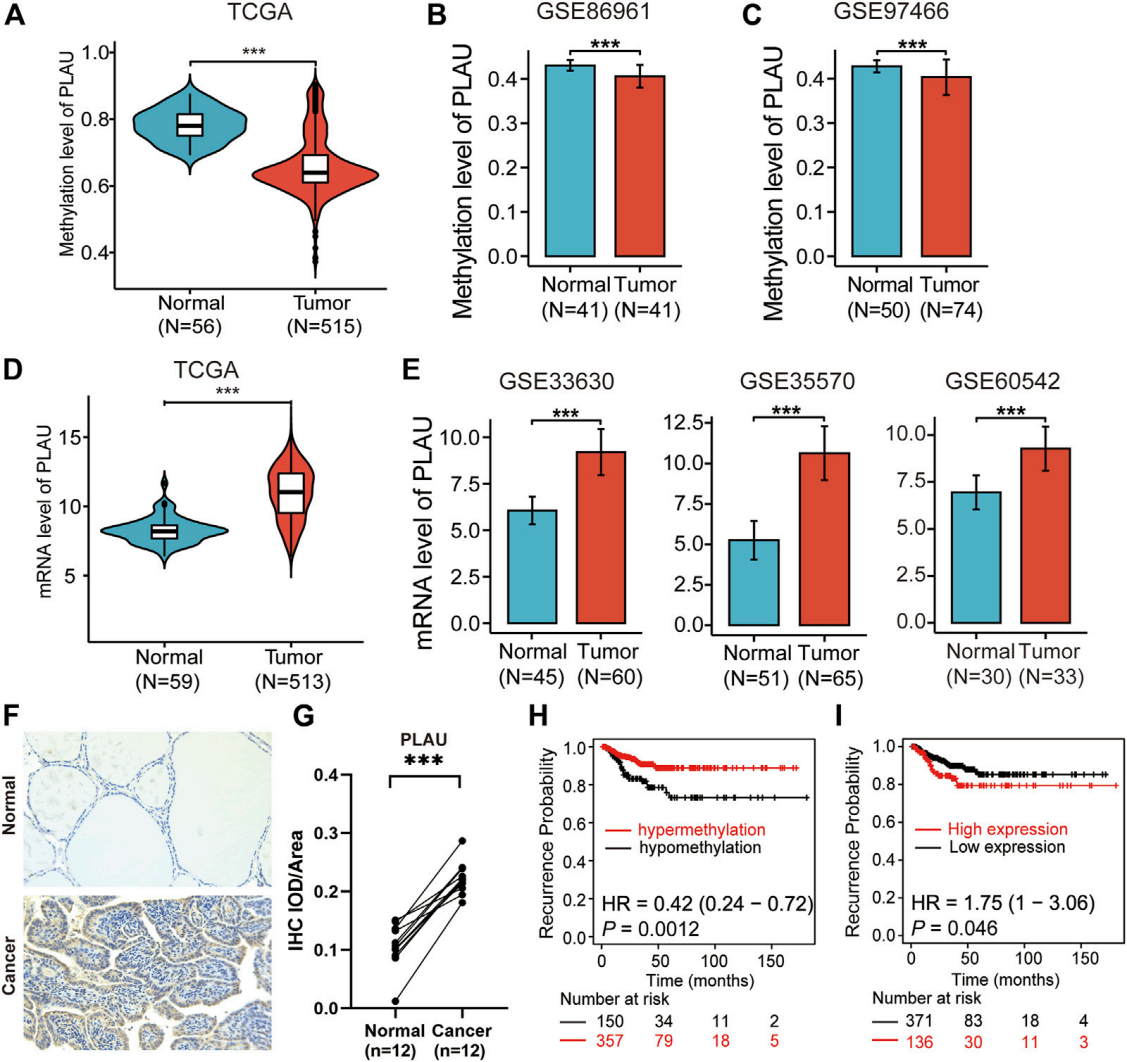
Validation of methylation levels and the expression of PLAU in THCA. Violin plots showing **(A)** the methylation levels and **(D)** mRNA levels of PLAU between normal and tumor tissues in THCA patients, Student’s t-test, ***p < 0.001. **(B,C)** Validation of the methylation levels of PLAU in THCA using data from the GEO database (GSE89961 and GSE97466), Student’s t-test, ***p < 0.001. **(E)** Comparison of the expression of PLAU between normal and tumor tissue in THCA patients using data from the GEO database (GSE33630, GSE35570 and GSE60542), Student’s t-test, ***p < 0.001. **(F,G)** Detection of the expression of PLAU in 12 pairs of matched cancerous and para-cancerous tissues of DTC via immunohistochemistry (IHC), Student’s t-test, ***p < 0.001. Kaplan-Meier plot for relapse-free survival (RFS) in THCA patients with different **(H)** methylation levels or **(I)** mRNA levels of PLAU.

As shown in Figure 3A, Figure 3H and Supplementary Figure S2, we observed that the PLAU methylation levels were reduced in thyroid cancer compared to normal thyroid tissue and were associated with a poor prognosis. Meanwhile, the transcription level of PLAU was higher in thyroid cancer, which indicates a poor prognosis (Figures 3D,I). Furthermore, the GSE89961 and GSE97466 datasets (Figures 3B,C) in the GEO data confirmed that the PLAU methylation levels in the tumor tissues were reduced compared with those in normal tissues, and GSE33630, GSE35570 and GSE60542 confirmed that the mRNA expression levels were increased (Figure 3E). Similarly, up-regulated expression was detected in 12 pairs of matched cancerous and para-cancerous tissues of DTC via immunohistochemistry (Figures 3F,G). Even when included in the expanded analysis of 337 normal samples from the GTEx database, our results suggested that PLAU still significantly overexpressed in tumor tissue (Supplementary Figure S3). Interesting, PLAU also functioned as oncogene in papillary and follicular thyroid cancers (Supplementary Figure S4).

### Highly Expressed PLAU Is Strongly Associated With a Poor Prognosis in DTC Patients

The demographic and clinical characteristics of the patients are listed in Table 1. We included 507 patients with 140 (27.61%) men and 367 (72.29%) women. The patients with age ≥55 years comprised 23.39%. The stage of the DTC patients ranged from I to IV according to the AJCC8th, and 402 (79.29%) patients were in stage I, 85 (16.77%) patients were in stage Ⅱ, 16 (3.16%) patients were in stage Ⅲ, and 4 (0.79%) were in stage IV. There were 229 (49.89%) and 285 (90.27%) patients with lymph node involvement or extrathyroidal extension, respectively. The pathologic stage was defined according to the American Joint Committee on Cancer (AJCC) staging manual. The histological types of the studied patients were TPC- Classical/usual 361 (71.20%), TPC- Follicular (≥99% follicular patterned) 102 (20.12%), and TPC-Tall cell (≥50% tall cell features) 44 (8.68%). The patients were divided into two groups based on BRAF mutation status: positive 157 (39.55%) and negative 240 (60.45%). The associations between the clinical variables, tumor specimens and the transcription level of PLAU were analyzed. We found that the transcription level of PLAU was closely related to age (*p* = 0.011) and stage (*p* < 0.001) based on the AJCC 8th, lymph node involvement (*p* < 0.01), histopathology (*p* < 0.001) and BRAF mutation (*p* < 0.01), Interestingly, the proportion of PLAU high expression was significantly increased in patients with lymph node involvement (35.4 vs 21.5%), while sex (*p* = NA) showed no association with the transcription level of PLAU. The number of DTC patients with extrathyroidal extension or distant metastases was limited, and no significant difference was observed. Overall, the results in Table 1 are all consistent with the correlation degree between PLAU methylation levels and the clinicopathological features shown in Figure 2D. Additionally, as shown in Supplementary Table S2 and Supplementary Table S3, stage and the expression of PLAU were found as the independent risk factors for recurrence of DTC patients.

**TABLE 1:** Correlation between PLAU expression and clinical characteristics in the TCGA cohort.

Variables	Total	Expression of PLAU		*p* Value
		High (*n* = 136)	Low (*n* = 371)	
Sex				
Female	367	98 (26.7)	269 (73.3)	NA
Male	140	38 (27.1)	102 (72.9)	
Age				
≥55	149	52 (34.9)	97 (65.1)	0.011
< 55	358	84 (23.5)	274 (76.5)	
AJCC stage 8th				
I	402	86 (21.4)	316 (78.6)	< 0.001
II	85	38 (44.7)	47 (55.3)	
III	16	11 (68.8)	5 (31.2)	
IV	4	1 (25.0)	3 (75.0)	
Lymph node involvement				
No	228	49 (21.5)	179 (78.5)	< 0.01
Yes	229	81 (35.4)	148 (64.6)	
Extrathyroidal extension				
No	285	85 (29.8)	200 (70.2)	NA
Yes	8	2 (25.0)	6 (75.0)	
Histopathology				
Classical	361	104 (28.8)	257 (71.2)	< 0.001
Follicular	102	6 (5.9)	96 (94.1)	
Tall Cell	44	26 (59.1)	18 (40.9)	
BRAF mutation				
Positive	157	26 (16.6)	131 (83.4)	< 0.01
Negative	240	75 (31.3)	165 (68.7)	

**FIGURE 4:**
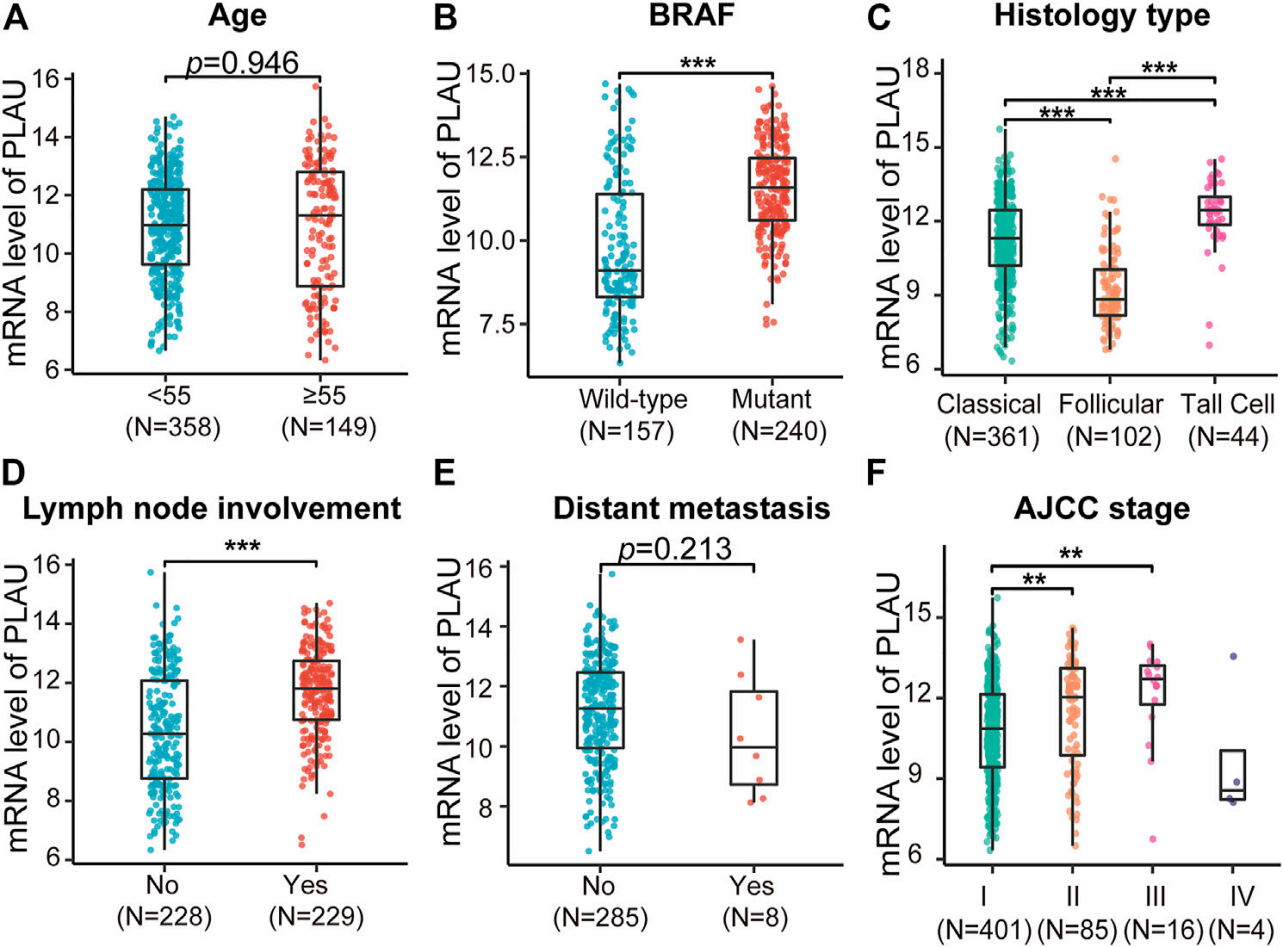
Comparative analysis of the mRNA levels of PLAU in different THCA subtypes. The expression data of PLAU and the matched clinical information were downloaded from the TCGA database. The mRNA level of PLAU was compared in THCA patients with different **(A)** age, **(B)** BRAF mutation status, **(C)** histological type, **(D)** lymph node metastasis status, **(E)** distant metastasis status, and **(F)** stage, Student’s t-test, **p < 0.01, ***p < 0.001.

In addition, based on the TCGA data, the statistically significant clinical characteristics obtained from the above results were divided into subgroups, and the PLAU transcription levels were compared. We found that the transcription level of PLAU between age < 55 and age >55 was not significantly different (Figure 4A). In addition, distant metastasis had nothing to do with the transcription level of PLAU, while the transcription level of PLAU was significantly increased in patients with BRAF mutations (Figure 4B) and lymph node involvement (Figure 4D). Among the pathological subtypes of DTC, the high-cell type had the highest transcription level, the classic type had the lowest, and the difference analysis was statistically significant (Figure 4C). In the AJCC stage I ∼ III subgroup analysis, with the increase in stage, the transcription level of PLAU was increased, and the results were statistically significant (Figure 4F).

### PLAU May Act by Participating in Immune-Related Pathways

**FIGURE 5:**
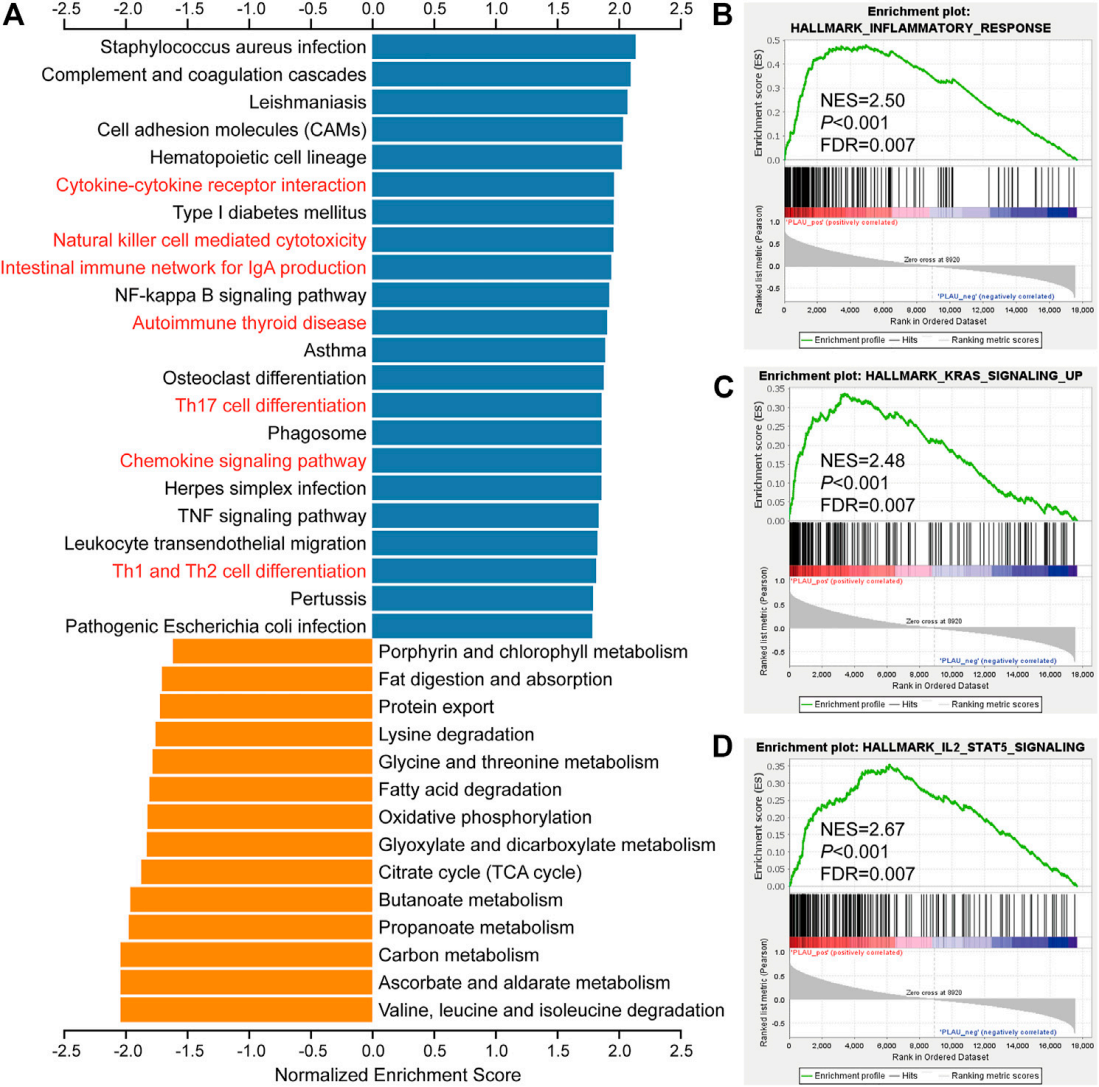
Biological function analysis of PLAU-related genes. **(A)** KEGG pathway analysis of PLAU-related genes. Up- and downregulated genes are indicated by blue and orange bars, respectively. **(B–D)** GSEA of differentially expressed PLAU-related genes, inflammatory response pathways, KRAS signaling upregulation pathways, and IL2/STAT5 signaling pathway were significantly enriched.

To clarify the role of PLAU in DTC, we further explored the functions of PLAU. We obtained PLAU- related differentially expressed genes (Supplementary Table S4) and further performed functional enrichment. The KEGG results showed that cytokine-cytokine receptor interaction, natural killer cell mediated cytotoxicity, intestinal immune network for IgA production, autoimmune thyroid disease, and Th17 cell differentiation were significantly upregulated (Figure 5A). GSEA suggested that the inflammatory response, KRAS signaling, and IL-2/stat5 signaling were upregulated (Figures 5B–D) in the PLAU high expression group. Similarly, KRAS and IL-2/stat5 signaling are involved in the regulation of DTC (Bos, 1989; Yoo et al., 2019; Al-Salam et al., 2020). Based on this perspective, the status of PLAU in THCA patients is important.

### Tumor Immune Cell Infiltration and Biomarkers in the DTC Immunological Microenvironment

**FIGURE 6:**
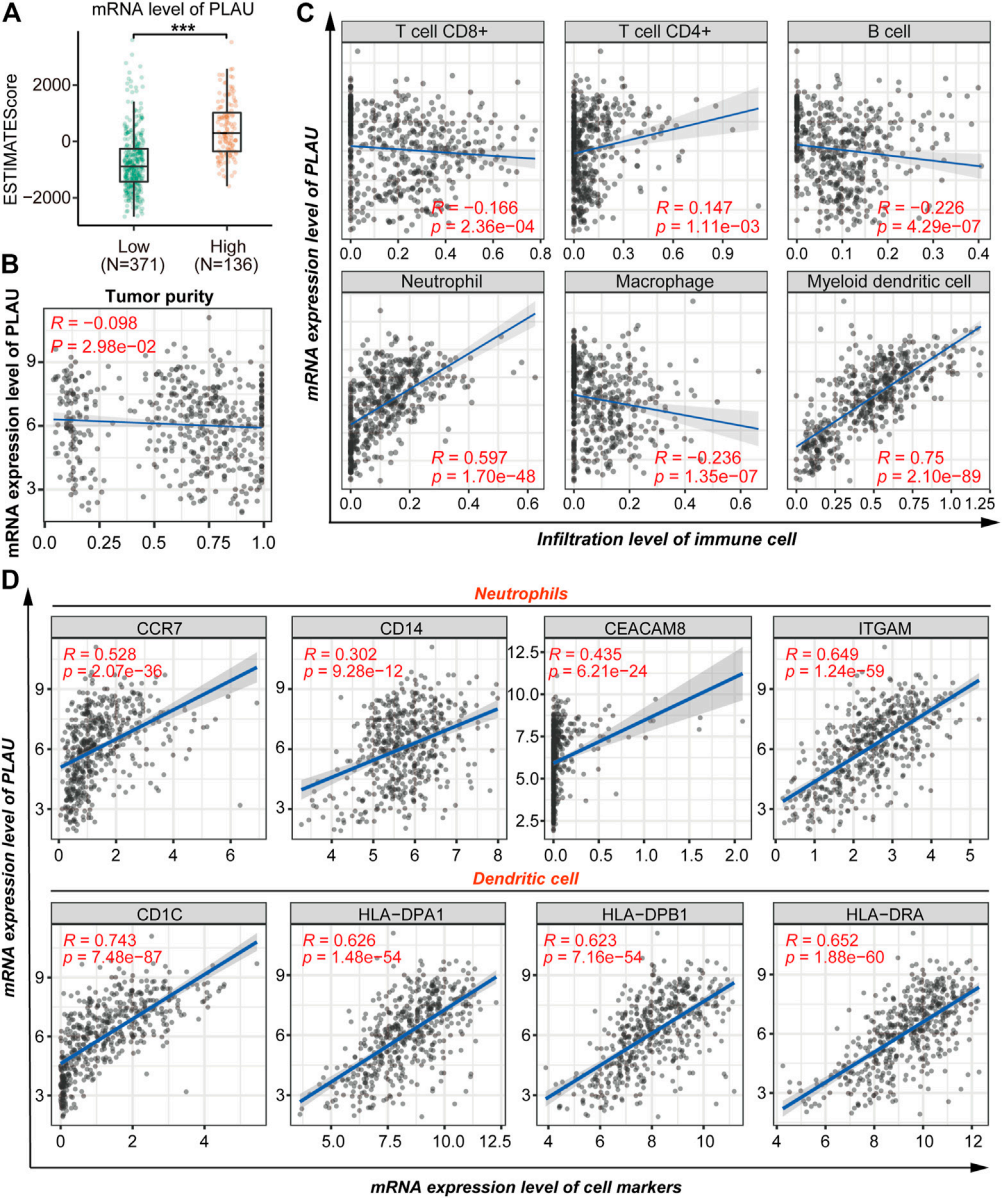
Correlation analysis of PLAU and immune cell infiltration. **(A)** Comparison of the immune scores (presented as the ESTIMATE Score) between THCA patients with different mRNA levels of PLAU, Student’s t-test, **p < 0.01, ***p < 0.001. **(B)** Correlation between the mRNA level of PLAU and tumor purity was evaluated using Pearson’s correlation test. **(C)** Pearson correlation was applied to analyze the correlation between the mRNA level of PLAU and the infiltration level of immune cells. **(D)** Pearson correlation was applied to analyze the correlation between the mRNA level of PLAU and the cell markers of the neutrophils and dendritic cells.

Given that the previous analysis suggested a possible role for PLAU in the immune response, in this section we explore the possible role of PLAU in the immune microenvironment of thyroid cancer. The results revealed a significant increase in the immunoscore of higher mRNA levels of PLAU (Figure 6A). Moreover, the transcription level of PLAU had nothing to do with the purity of the tumor (Figure 6B). Tumor-infiltrating lymphocytes are an independent predictor of immune surveillance in the prognosis of various cancers. Therefore, we further explored whether the mRNA expression of PLAU was correlated with the level of immune infiltration in THCA. The results indicated that PLAU was positively correlated with CD4^+^ T cells (R = 0.147, *p* = 1.11e-03), neutrophil (R = 0.597, *p* = 1.70e-48), myeloid dendritic cell (R = 0.75, *p* = 2.10e-89) immune infiltration levels in THCA (Figure 6C).

Subsequently, we explored the relationships between the transcription level of PLAU and immune marker genes of different immune cells in the TIMER database for further validation, including neutrophils, dendritic cells, B cells, CD8^+^ T cells, Th1 cells, Th2 cells, Th17 cells, and Treg cells in THCA (Supplementary Table S1). Interestingly, we found that the expression levels of most immune markers were significantly correlated with the transcription level of PLAU (Figure 6D). Tumor infiltration and the expression of immune markers of neutrophils and dendritic cells showed a significant positive correlation with PLAU.

### Aberrant Methylation of cg06829584 Leads to the Upregulation of PLAU in DTC

**FIGURE 7:**
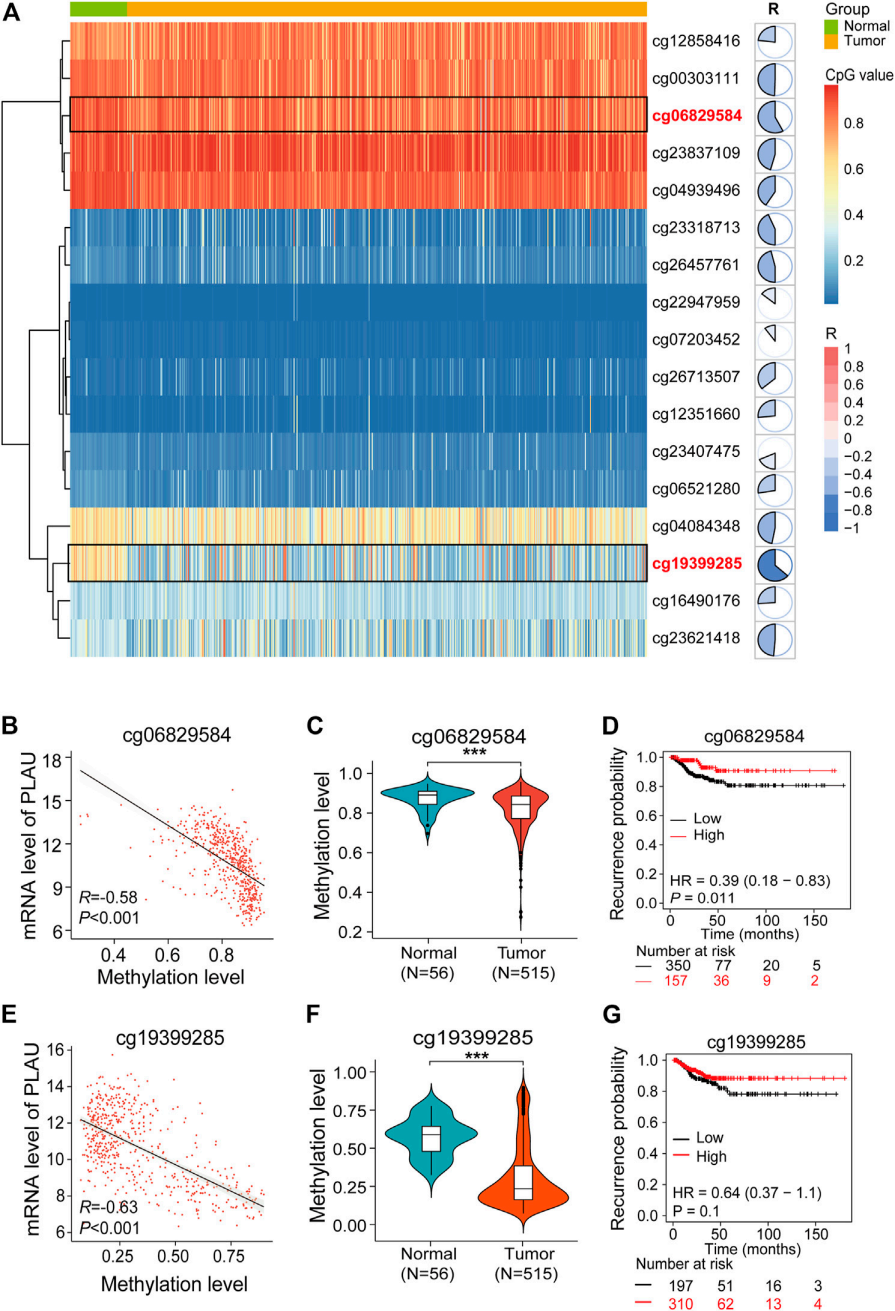
Aberrant methylation of cg06829584 results in the upregulation of PLAU in THCA. **(A)** Heatmap showing the expression of different probes in THCA. Pie charts show the correlation between the expression of probes and PLAU. **(B,E)** Pearson correlation was applied to analyze the correlation between the mRNA level of PLAU and the methylation level of the cg06829584 and cg19399285 probes. Violin plots showing the methylation levels of the cg06829584 **(C)** and cg19399285 probes **(F)** between normal and tumor tissues in THCA patients, Student’s t-test, ***p < 0.001. Kaplan-Meier plot of the relapse-free survival (RFS) in THCA patients with different methylation levels of the cg06829584 **(D)** and cg19399285 probes **(G)**.

The heatmap shows the degree of methylation of different probes in normal and tumor tissues, and the pie charts quantify the degree of correlation between the level of probe methylation and the PLAU transcription level (Figure 7A). We found that the expression of the cg06829584 and cg1939925 probes between the normal and tumor tissues was significantly different (Figure 7A).

Finally, we examined the correlation between the cg06829584 and cg1939925 probes and the PLAU transcription level, and the results showed that cg06829584 (R = −0.58, *p* < 0.001) and cg1939925 (R −0.63, *p* < 0.001) were negatively correlated with the PLAU transcription level (Figure 7B). Compared with normal tissues, the methylation levels of the above two probes in tumor tissues were significantly lower (Figures 7C,F), which was implicated in a worse prognosis (Figures 7D,G).

## Discussion

From 1994 to 2013, the overall incidence of thyroid cancer in the United States increased markedly (approximately 3% per year) (Lim et al., 2017), and during this period, a sharp increase in the thyroid cancer mortality rate was also reported (approximately 1.1% annually), especially in advanced-stage PTC (2.9% per year) (Lim et al., 2017). In fact, although most patients with thyroid cancer have a good prognosis, some patients have a poor prognosis due to tumor recurrence or distant metastasis (Kazaure et al., 2012). Therefore, exploring new biomarkers is of great significance and can help identify patients with a poor prognosis.

The role and potential therapeutic and diagnostic value of methylation-related genes in thyroid cancer have been well recognized. In this study, we found that methylation-driven genes were closely associated with the prognosis of DTC, and we reported for the first time that abnormal expression of methylation-driven PLAU genes was an independent risk factor for recurrence in DTC patients. This result provides a new idea for the prognostic management of thyroid cancer patients.

PLAU, a type of serine protease, can convert plasminogen to plasmin, which plays an important role in degrading the extracellular matrix and promoting fibrinolysis (Sudol, 2011). PLAU and PLAUR, members of the PLAU system, are overexpressed in several malignant tumors and are associated with the complex phenotype of cancer. Therefore, the PLAU system has been widely explored as a target for anticancer drug therapy (Ulisse et al., 2009; Hildenbrand et al., 2010). Gabriel found that PLAU was involved in the metastasis of invasive breast cancer, and had an unexpected complexity of transcription (Moquet-Torcy et al., 2014). Overexpression of PLAU significantly promoted the proliferation, migration and angiogenic abilities of colorectal cancer cells (Lin et al., 2019). Previous studies have shown a significant increase in uPA mRNA and activity in differentiated thyroid carcinoma cell lines (Ulisse et al., 2006), which is consistent with our research conclusion.

In the current study, upregulation of the PLAU gene was associated with increased recurrence in DTC patients. The current consensus is that an old age (>45), extrathyroidal extension, vascular invasion, or metastases are considered to be independent risk factors for PTC RFS (Haugen et al., 2016; Grønlund et al., 2021). Moreover, we found that high expression of PLAU was associated with lymph node metastasis, and the potential mechanism was an immune-related pathway, which means that PLAU gene expression analysis helps predict the prognosis and survival of THCA patients.

Many studies have suggested that autoimmunity and inflammation are risk factors for thyroid cancer (Ferrari et al., 2020). The “neutrophil-to-lymphocyte ratio” (NLR) has been proven to be associated with tumor progression: an elevated NLR is correlated with a larger tumor size and a higher risk of recurrence (Bhatti et al., 2010). In human TC samples, neutrophil density is correlated with the tumor size, suggesting a potential tumor-promoting role of tumor-associated neutrophils (Galdiero et al., 2018). Our study likewise demonstrated that neutrophil infiltration was positively correlated with PLAU expression and was associated with a poor prognosis (Ferrari et al., 2019). In addition, CD8^+^ T cells kill PTC tumor cells, and PLAU expression was negatively correlated with CD8^+^ T cell infiltration, suggesting a poor prognosis, which is also consistent with our findings (Yin et al., 2020).

Moreover, a pan-cancer analysis revealed that high expression of the fibrinolysis gene clusters PLAU, PLAUR, and SERPINE1 was consistently correlated with a high expression of tumor microenvironment (TME) monocyte infiltration features and important checkpoints of the immune response, such as PD-L2 and CD276/B7-H3 (Saidak et al., 2021). The PLAU expression product uPA can induce/attenuate the immune inflammatory response through AMPK signaling and the PI3K/Akt pathway, which is important in the development of thyroid cancer (Dinesh and Rasool, 2018). In addition, some studies have found that augmented expression of uPA and uPAR at both the mRNA and protein levels is associated with the malignant transformation of human thyroid cells. More importantly, the increased expression of uPA in PTC tissues is associated with an aggressive, advanced stage and a shorter disease-free interval of tumors (Ulisse et al., 2006; Baldini et al., 2012). In addition, uPA, the serine protease encoded by the gene PLAU, can activate plasminogen, which degrades fibrin (Saidak et al., 2021). The TME contains multiple cell types that interact with each other and contribute to the tumor ecosystem (Binnewies et al., 2018). Coagulation and fibrinolysis are regulated by inflammation and local leukocyte recruitment in the TME (Foley and Conway, 2016; Date et al., 2017). Previous studies have confirmed a positive association between the expression of immune genes and the fibrinolysis gene PLAU. For PLAU, the strongest association was observed for the PDCD1LG2 and CD276 genes, which encode the checkpoints PD-L2 and B7-H3 (Saidak et al., 2021).

Thus, mechanistically, PLAU is involved in the regulation of pathways related to the immune microenvironment of thyroid cancer, and it is closely associated with the development of thyroid cancer.

## Conclusion

In conclusion, upregulation of the PLAU gene is associated with an increased risk of recurrence by interfering with immune pathways in THCA patients. Hypomethylation of cg06829584 and cg1939925 leads to high expression of PLAU in DTC patients. PLAU can be considered a potential biomarker for prognosis and immune-related therapeutic targets in THCA patients.

## Data Availability

The datasets presented in this study can be found in online repositories. The names of the repository/repositories and accession number(s) can be found in the article/Supplementary Material.
