# Supplementary figures and images for: Methylation-Driven Gene PLAU as a Potential Prognostic Marker for Differential Thyroid Carcinoma
Source: Front Cell Dev Biol. 2022 Jan 24;10:819484. doi: 10.3389/fcell.2022.819484 (PMC8818873; doi:10.3389/fcell.2022.819484)

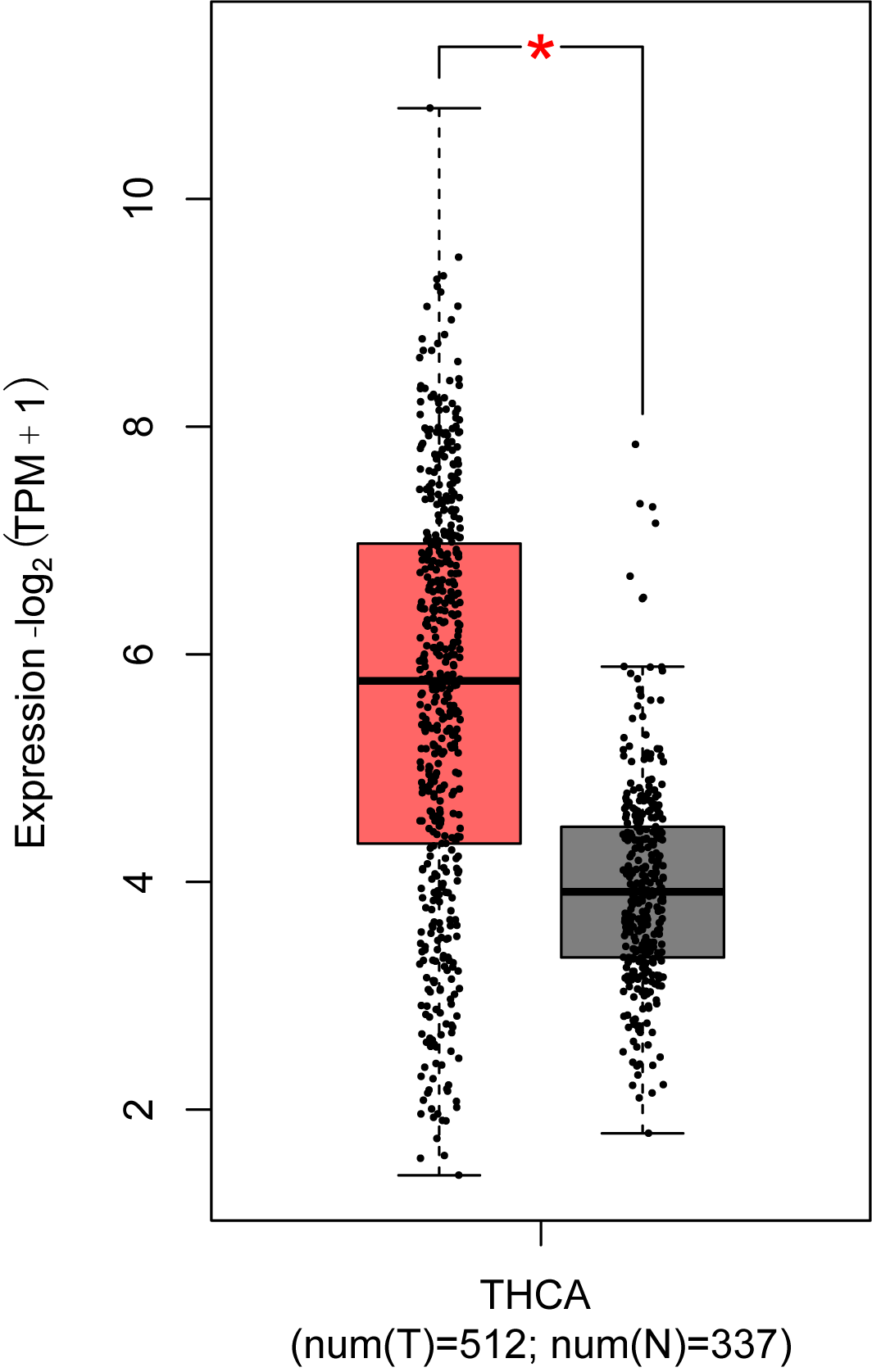

Supplement: Supplementary file 3 [file Image3.TIF]

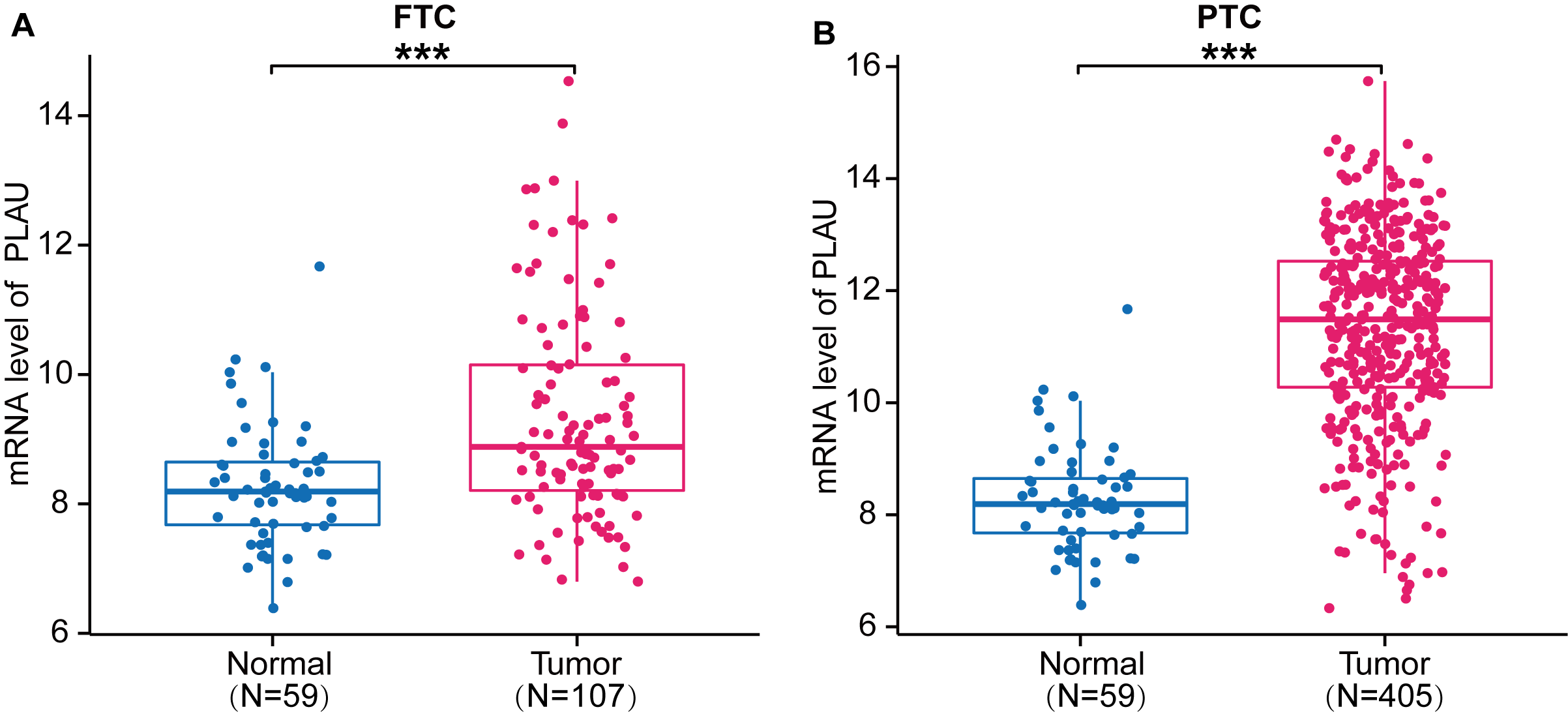

Supplement: Supplementary file 4 [file Image4.TIF]

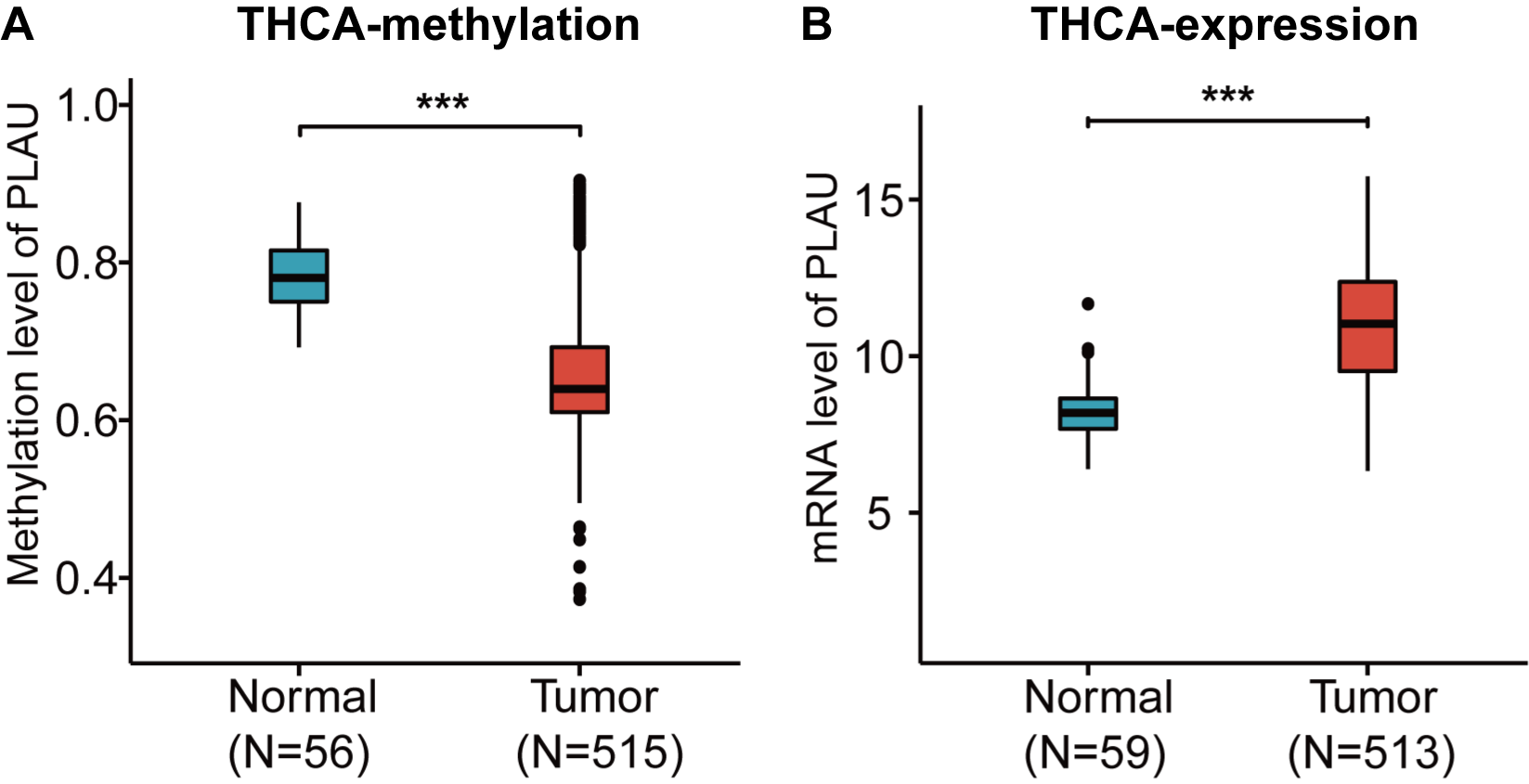

Supplement: Supplementary file 5 [file Image2.TIF]

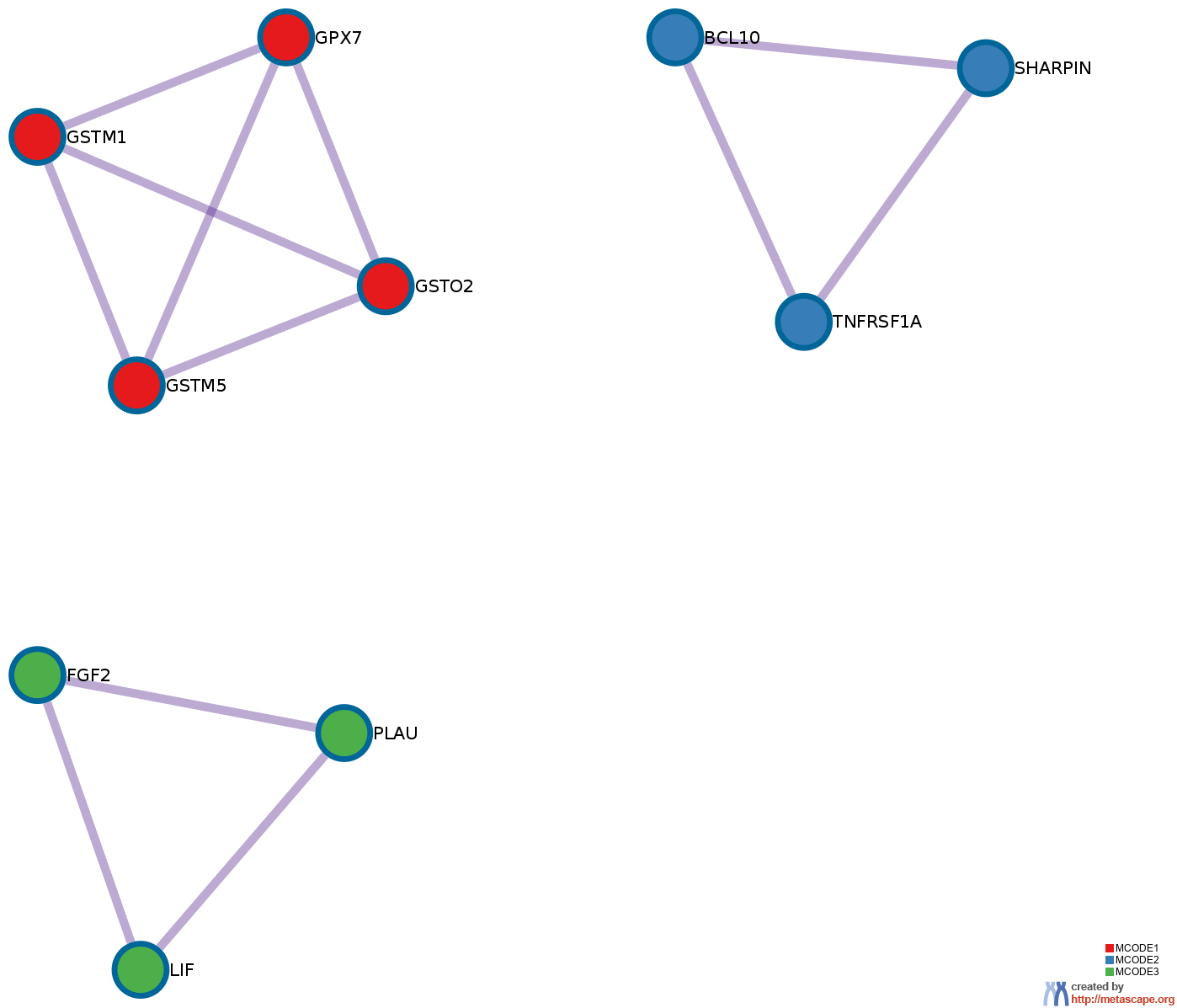

Supplement: Supplementary file 6 [file Image1.TIF]
